# A Clinical Case of Three-Dimensional Electrical Impedance Tomography (3D-EIT) Measurements

**DOI:** 10.7759/cureus.73291

**Published:** 2024-11-08

**Authors:** Yoshiaki Iwashita, Shun Takeda, Satoshi Kawashima, Tomoya Koshi, Ken-ichi Nomura, Shinya Sato, Rie Sato, Noriaki Yamada, Satoru Nebuya

**Affiliations:** 1 Department of Emergency and Critical Care Medicine, Shimane University, Izumo, JPN; 2 Department of Development, Posh Wellness Laboratory Inc., Tokyo, JPN; 3 Sensing System Research Center, National Institute of Advanced Industrial Science and Technology, Tsukuba, JPN; 4 Department of Rehabilitation Medicine, Shimane University Hospital, Izumo, JPN; 5 Department of Joint Research in Advanced Medicine for Electromagnetic Engineering, Shimane University, Izumo, JPN

**Keywords:** belt, electrical impedance tomography, pneumonia, three-dimensional, wearable biosensing devices

## Abstract

Electrical impedance tomography (EIT) is an emerging imaging modality that assesses the bioelectrical impedance of the chest wall. It enables the generation of immediate topographical images non-invasively and without the use of radiation. However, current commercial systems are limited to single-plane measurements. Recently, we developed a prototype of a multi-slice EIT system that can reconstruct data into three-dimensional (3D) EIT images. Here, we report the first case of using 3D-EIT measurements in a patient diagnosed with severe pneumonia.

A 62-year-old female presented with fever, cough, and difficulty in breathing. The patient was diagnosed with bacterial pneumonia in her bilateral lower lobes based on chest radiography, CT, and laboratory data. The patient’s profile was as follows: height, 160 cm; weight, 52 kg; and chest height, 84 cm. The 3D-EIT system comprised eight electrodes per slice surface, with four fault lines positioned on a single belt. We successfully obtained four EIT slices simultaneously, which were converted to 3D-EIT representations. These four slices were almost identical to those in the CT scans. Notably, in this patient with lower-lobe pneumonia, the 3D-EIT system revealed that the air entered the upper area first, whereas the healthy volunteer breathed in the lower lobe first.

We successfully performed 3D-EIT in patients with pneumonia. Specifically, in lower-lobe pneumonia cases, our findings showed a reversed airflow pattern, with air initially entering the upper area. This underscores the potential of our developed technology to offer novel insights into the vertical sequence of air inflow.

## Introduction

Electrical impedance tomography (EIT) is a relatively new imaging technique that enables cross-sectional tomography devoid of radiation exposure. The principle of the measurement was as follows: 8-32 electrodes were attached horizontally and evenly spaced across the chest. A weak current was applied between two adjacent points, allowing the measurement of the body’s biological impedance, from which a tomographic image was generated based on the results and electrical characteristics. The greatest advantage of EIT lies in its capacity to capture dynamic images, leading to the discovery of phenomena such as pendelluft, which is not observable in other imaging modalities [1]. Despite the availability of several machines commercially, current existing systems are limited to single-plane measurements. Recent studies have introduced the possibility of obtaining multi-slice EIT data, which can be converted into 3D-EIT representations [2-5]. Recently, we developed a prototype of a multi-slice EIT system capable of reconstructing data into 3D-EIT. Here, we report the first clinical case of 3D-EIT measurements in a patient with severe pneumonia.

## Case presentation

**Figure 1:**
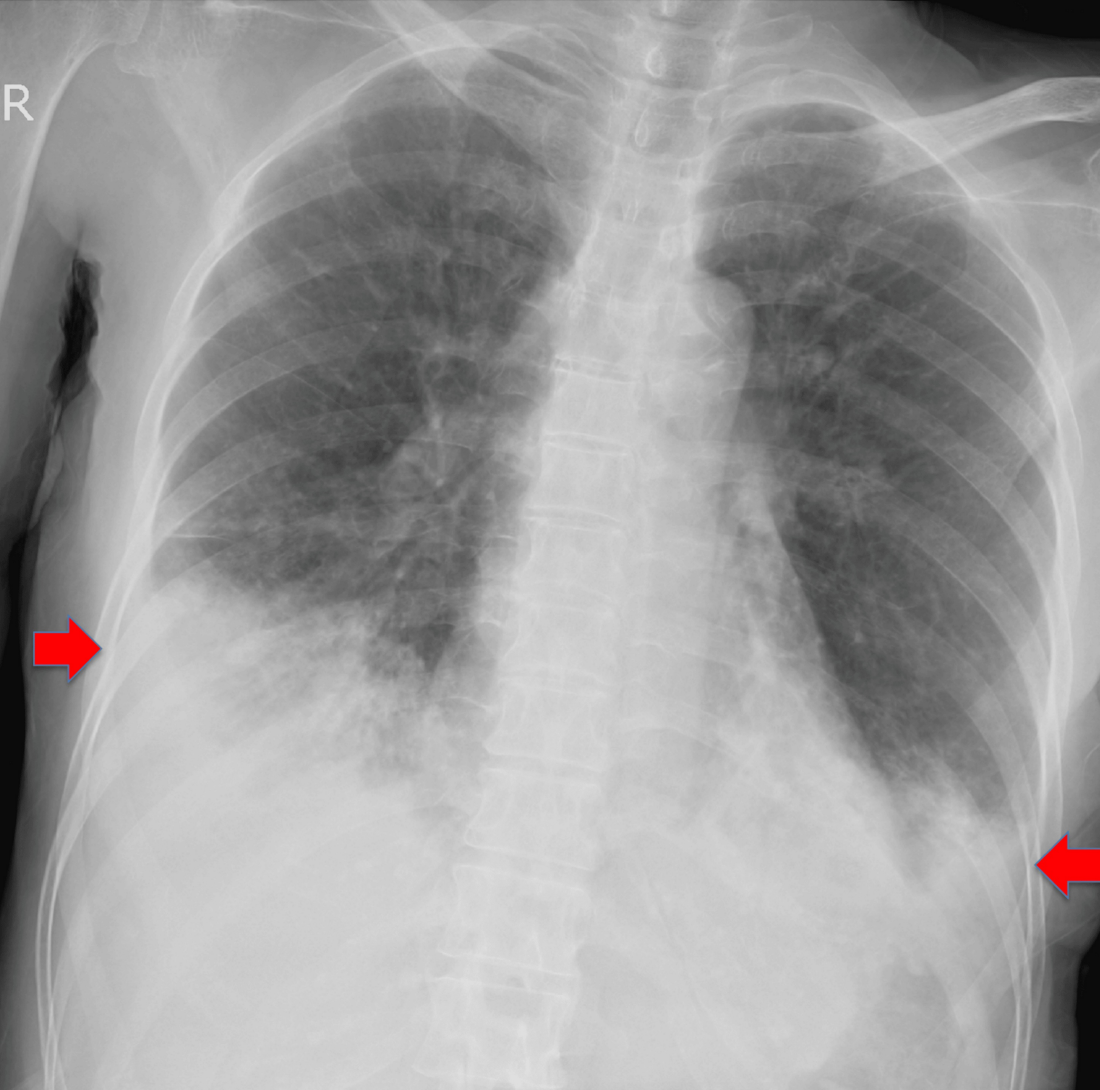
Chest X-ray of the patient Bilateral lower area consolidation is seen (red arrows).

**Figure 2:**
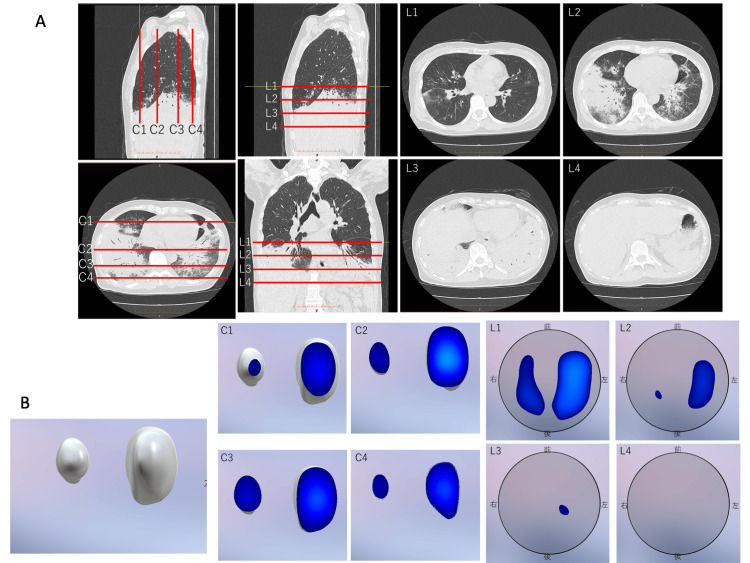
(A) Chest CT of the patient; (B) Chest EIT of the patient. L1 to L4 indicates the position of the scanned layer of EIT. C1 to C4 indicates the coronal section of the EIT scan. EIT: electrical impedance tomography

A 62-year-old female presented with fever, cough, and difficulty in breathing, with symptoms manifesting seven days prior to admission and worsening over time. She had a history of untreated asthma. On examination, the results showed temperature - 38.1°C, SpO2 - 93% on room air, respiratory rate - 22/min, blood pressure - 124/72, and heart rate - 92/min. Laboratory data revealed a marked increase in inflammatory responses (white blood cell (WBC) 25000/µL, C-reactive protein (CRP) 44.9 mg/dL). Chest radiography revealed bilateral lower lobe consolidation (Figure 1), while the chest CT scans showed marked pneumonia (Figure 2A). COVID-19 polymerase chain reaction (PCR) test results were negative, leading to a diagnosis of bacterial pneumonia. Following the diagnosis and antibiotics administration, we obtained informed consent for the application of 3D-EIT.

**Figure 3:**
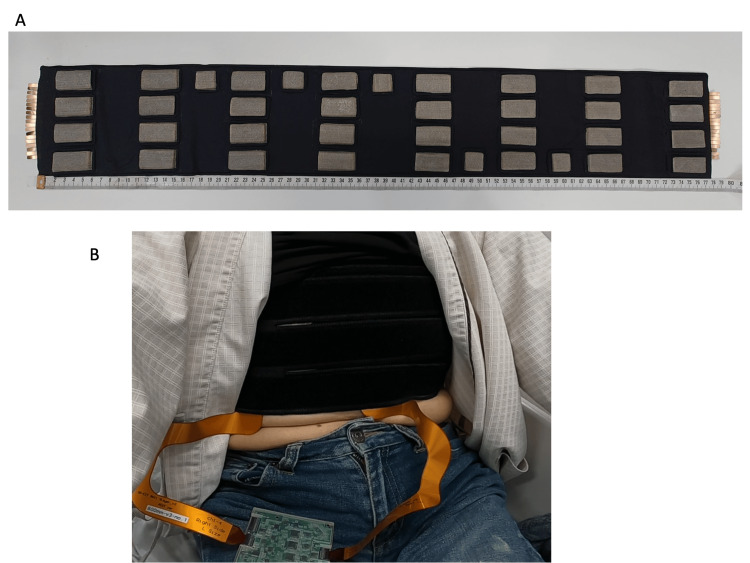
Photograph of the belt (A) and the belted patient (B). Figure 3A shows the 3D-EIT belt and Figure 3B shows the overview of the patient wearing the belt. EIT: electrical impedance tomography

The patient’s profile was as follows: height, 160 cm; weight, 52 kg, and chest height, 84 cm. Figure 3A shows an overview of the wearable 3D-EIT belt we have developed. The belt was stretchable and comprised a rubber belt, meander wiring, textile-based conductive electrodes, and a measurement circuit. The measurement circuit was set on the belt, which was 800-1000 mm in length and 140 mm in width, and included eight four-layer electrode wires. The electrodes were made of silver-coated thread, and their dimensions were set to 40 mm width, 20 mm length, and 15 mm height. The electrodes were equally spaced to reconstruct the EIT images using back projection. The total weight of the wearable EIT belt, including that of the measurement circuit, was approximately 300 g.

**Figure 4:**
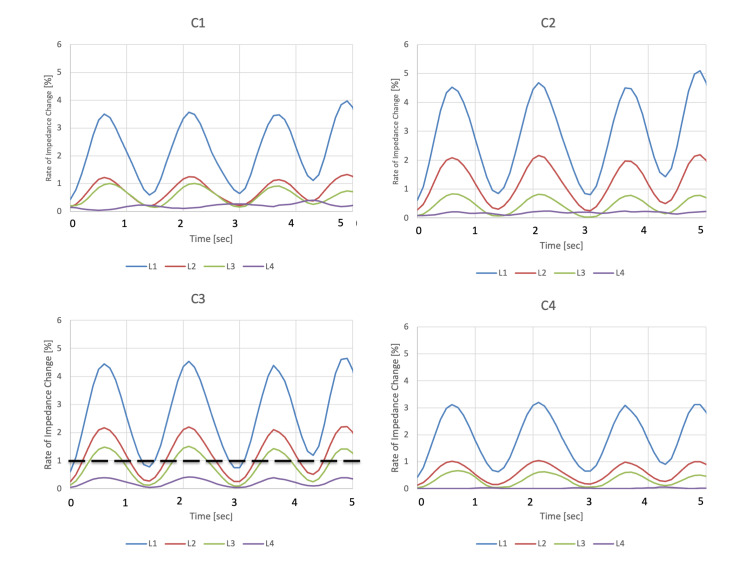
Time course of the coronal view of EIT scan. Air inflows to the upper side first and spread to the lower side. EIT: electrical impedance tomography

The measurement circuit was controlled by a personal computer (PC) through a USB cable. The impedance measurement circuitry measures the complex impedance by amplifying the difference between voltages at adjacent receiver electrodes. The complex transfer impedances were calculated across frequencies ranging from 10 kHz to 200 kHz. Eight of these were used in the circuit. To minimize the number of components, only one drive-circuit unit is used to apply a current of 1 μA. The constant current circuit used a bipolar drive and had an output impedance of 200 kΩ at a frequency of 200 kHz. All units were controlled using a microcontroller via a serial peripheral interface (SPI). Using high-speed analog switches, eight-by-four-layer electrode measurements at a single frequency were made at frame rates of 12 (frame/s). Figure 3B shows an overview of a patient wearing the 3D-EIT shirt. The belt was placed on as upper chest as possible avoiding interference with the breast. 3D views of the EIT and axial sections per slice are shown in Figure 2B. C1 to C4 indicates the coronal view of the EIT scan and L1 to L4 indicates the axial view of the EIT scan. On visual inspection, the CT and EIT modalities seem to capture roughly the same anatomical features. Figure 4 shows the time course of the rate of impedance change in axial view, revealing consistent airflow patterns where air initially entered L1, followed by L2 and L3, indicating a superior to inferior airflow sequence in coronal views.

## Discussion

In this clinical case, we obtained 3D-EIT images, which were subsequently compared with the CT images, and were assessed by two physicians as accurately imaged on visual inspection.

Currently, EIT is used in medical practice to detect atelectasis and pneumothorax [6], evaluate alveolar recruitment [7], and determine the appropriate positive end-expiratory pressure (PEEP) settings [8]. It has also been used to detect the pendelluft phenomenon, a phenomenon of asynchronous inspiration [1]. In particular, the pendelluft phenomenon was discovered because of the dynamic imaging capability of EIT and would have otherwise remained undetectable with conventional static imaging modalities. Currently, there are several EIT devices on the market. Most use 16-32 electrodes per slice, resulting in resolutions of 16 × 16 or 32 × 32 pixels, which are significantly lower than that of CT (512 × 512 pixels per slice). Since the clinical application of using EIT is to set PEEP or to detect the pendelluft phenomenon, we believe that detailed imaging is not necessarily needed. Instead, obtaining information on dynamic air inflow across multiple slices, including the vertical direction, rather than obtaining high-resolution images in a single slice proves beneficial. Thus, we developed a belt capable of providing four cross-sectional images of the eight electrodes per slice.

Multi-slice EIT has been reported several times at the research level, with various methods proposed for multi-slice 3D reconstruction, including measurements and simulations using phantom models [2-4]. Grychtol et al. simulated two planes using 16 electrodes EIT in horses and human volunteers [5]. In the present case of extensive lower-lobe pneumonia, air inflow was found to enter from the bilateral upper areas. This contrasts with our prior measurements in healthy volunteers, in which the air inflow came from the bilateral lower areas. Although the clinical consequences related to the air inflow sequence in this present case report are unclear, it is worth noting that no other device can measure the sequence of air inflow in the vertical direction in real time. This presents an opportunity for new discoveries in the future.

## Conclusions

We have successfully measured 3D-EIT in a patient with lower lobe pneumonia. Air flows into the upper area first. This suggests that the technology could provide new information about the sequence of air inflow in the vertical direction.
